# Behavioural osmoregulation during land invasion in fish: Prandial drinking and wetting of the dry skin

**DOI:** 10.1371/journal.pone.0277968

**Published:** 2022-12-07

**Authors:** Yukitoshi Katayama, Takehiro Tsukada, Susumu Hyodo, Hirotaka Sakamoto, Tatsuya Sakamoto

**Affiliations:** 1 Ushimado Marine Institute, Faculty of Science, Okayama University, Setouchi, Okayama, Japan; 2 Department of Biomolecular Science, Toho University, Funabashi, Chiba, Japan; 3 Laboratory of Physiology, Atmosphere and Ocean Research Institute, University of Tokyo, Kashiwa, Chiba, Japan; The University of Tokyo, JAPAN

## Abstract

Osmoregulatory behaviours should have evolutionarily modified for terrestrialisation of vertebrates. In mammals, sensations of buccal food and drying have immediate effects on postprandial thirst to prevent future systemic dehydration, and is thereby considered to be ‘anticipatory thirst’. However, it remains unclear whether such an anticipatory response has been acquired in the non-tetrapod lineage. Using the mudskipper goby (*Periophthalmus modestus*) as a semi-terrestrial ray-finned fish, we herein investigated postprandial drinking and other unique features like full-body ‘rolling’ over on the back although these behaviours had not been considered to have osmoregulatory functions. In our observations on tidal flats, mudskippers migrated into water areas within a minute after terrestrial eating, and exhibited rolling behaviour with accompanying pectoral-fin movements. In aquarium experiments, frequency of migration into a water area for drinking increased within a few minutes after eating onset, without systemic dehydration. During their low humidity exposure, frequency of the rolling behaviour and pectoral-fin movements increased by more than five times to moisten the skin before systemic dehydration. These findings suggest anticipatory responses which arise from oral/gastrointestinal and cutaneous sensation in the goby. These sensation and motivation seem to have evolved in distantly related species in order to solve osmoregulatory challenges during terrestrialisation.

## Introduction

Independently of the tetrapod lineage including lobe-finned fish, ray-finned fish have expanded their habitats from aquatic to terrestrial environments [1–3]. For example, *Periophthalmus* mudskipper gobies spend most of their lives on tidal flats and have physiological specialisations associated with a semi-terrestrial lifestyle [4]. Their adaptation to a terrestrial lifestyle also requires transformations in osmoregulatory behaviours to counter dehydration [5]. Indeed, *P*. *modestus* store water in the buccal and opercular cavities as a source of drinking water when on land [6]. Furthermore, our comparison of hormonal regulation of drinking behaviours among the *Periophthalmus* mudskipper and mammals suggested repeated evolution of thirst in distant taxa of vertebrates [6, 7]. As well as thirst regulated by hormones, postprandial thirst serves to prevent systemic dehydration in tetrapods [8, 9]. In postprandial thirst in mammals, buccal sensation of food/water has immediate effects on neural activities at the brain thirst center before any changes in blood parameters (e.g., osmolality, hormones); thereby, this phenomenon is considered to be ‘anticipatory thirst’. Although prandial drinking was shown also in birds [10], its anticipatory function has not been demonstrated. Therefore, it remains unknown how possible anticipatory behaviours derived from peripheral sensation have evolved.

On tidal flats, carnivorous mudskippers such as the *Periophthalmus* species eat a variety of prey, including marine invertebrates and terrestrial insects [11]. Their hunting can be observed clearly as a bending of the body followed by biting in the mud [12]. Migration of mudskippers to a tide pool is frequently observed after eating (see S1 Movie in our previous report [13]). This implies that buccal sensation of food or drying motivates mudskippers to move to water for drinking. Because mudskippers cannot absorb water through the cutaneous skin, unlike ‘cutaneous drinking’ in amphibians [14], analyses of their migration have been used as an index of their desire for water [6, 7]. Although the migration is triggered by many behavioural/physiological requirements [5], this index can allow examination of whether postprandial drinking has been acquired during terrestrialisation of ray-finned fish as an anticipatory response to potential dehydration.

Another potential osmoregulatory behaviour is observed on tidal flats: rolling over onto the dorsal surface and subsequent returning to their normal position. This is defined as ‘rolling behaviour’ [13] and has been suggested to moisten the dorsal skin, which is not keratinized [15]. However, it is unclear if this behaviour arises from cutaneous drying or other stimuli, such as systemic/blood parameters for osmoregulation.

The main aim of this study is to explore postprandial drinking and rolling behaviour in the semi-terrestrial mudskipper (*P*. *modestus*), which is independent of the tetrapod lineage, as possible anticipatory responses to potential dehydration. We analysed these behaviours in aquarium experiments, following our field observations, to examine the role of peripheral (buccal and cutaneous) hydromineral sensing in their osmoregulation.

## Materials and methods

### Field observation

Behaviour of each mudskipper in an estuary (35° 60’ N, 139° 55’ E) at low tide was recorded with a video camera (HC-VX985M, Panasonic, Japan) from May to June (e.g., rolling behaviour in S1 Movie [13]). No permits of the field site access were required because the site is not a sanctuary. For analysis of prandial drinking, 5-min movies were randomly selected. Mudskipper terrestrial eating is clearly observed because the fish put its mouth on the mudflat following bending of the body axis. Migration to water areas where mudskippers fully bathed their mouths was counted as an index of a desire to drink. The timing of bouts of terrestrial eating and those of migration to water areas was analysed to measure the latency of migration into a water area after terrestrial eating (see S1 Fig). A movie showing pectoral-fin movements was used to capture sequential images (Fig 4A).

### Experimental animals

One-year-old mudskippers of both sexes (*P*. *modestus*) weighing 3 to 5 g were collected from an estuary (34° 60’ N, 134° 05’ E). Plasma ions, differentiation of osmoregulatory organs, hormonal status, and amphibious behaviours (i.e., voluntary migration out of and into water) were examined in the mudskippers under varying conditions. Since we previously showed that there are no sex differences in the amphibious behaviour [16–18], both sexes of fish were used. Fish were acclimated for 2 to 5 weeks in laboratory tanks (3 L). Since these fish were collected from brackish water, the tank water was diluted seawater (10 ppt, 149 mM Na^+^, 176 mM Cl^−^, 3.8 mM Ca^2+^, 346 mOsml/kg), which is almost isotonic to mudskipper plasma. All fish were maintained at room temperature of 22–25°C under a daily photoperiod cycle of 12-h light/12-h dark (lights on at 7:00 a.m.) and were fed daily with Tetrafin flakes (TetraWerke, Melle, Germany). The mudskippers were provided with small plates to climb on in each tank. Before handling or sacrifice, fish were deeply anesthetized with 0.01% tricaine methanesulfonate (Sigma, Tokyo, Japan) neutralized with sodium bicarbonate. All experiments were approved by Dr. Matsukawa at the Animal Experiment Committee of Okayama University and Dr. Saito at the University of Tokyo, and were performed in accordance with manuals prepared by these committees.

### Testing for postprandial amphibious/drinking behaviour in aquaria

Intact fish (*n* = 6) fasted for 48 h were randomly selected and each fish was transferred to an experimental tank without food in the 2–9 h after light onset. A tank measuring 250 × 150 × 250 mm (L × W × D) (volume of 10-ppt diluted seawater 6000 mL) was used [6, 17, 18]. The land area was made of plastic mesh, and care was taken to ensure that there was minimum water on this area. Water in the tank was constantly aerated. The period in water and the frequency of migration between water and land area (defined as the ‘frequency of migration’) were recorded for 30 min as reported in our previous studies [6, 7, 17, 18]. After 30-min acclimation, amphibious behaviour was tested (‘before feeding’). Subsequently, 0.1 g/g body weight of Tetrafin was placed on a cotton plate on the land area without disturbing fish. In order to avoid their eating in the water area, Tetrafin flakes (TetraWerke, Melle, Germany), which often stick the wet skin of mudskippers, were pulverized in advance. Fish started eating in 30 min after the feeding. Immediately, the amphibious behaviour was tested (‘while eating’).

Based on the results of this observation, a follow-up experiment was performed using other fish. Fish (*n* = 7) fasted for 48 h were randomly selected for each experimental group (fed vs. unfed). Amphibious behaviour was similarly tested and analysed before feeding and every 5 min after eating onset. We also examined whether fluctuation of plasma sodium occurred at the time when the frequency of migration and period in water increased. Five minutes after eating onset, using other fish (*n* = 5), blood samples were quickly collected from the hemal arch in the region of the caudal peduncle as previously described [17, 19, 20]. Sodium ion concentration in their plasma was measured with an atomic absorption spectrophotometer (Z5300, Hitachi, Tokyo, Japan). The amount of drinking was also measured after 30 min. Fish were sacrificed after deep anesthesia and the amount of water in the gastrointestinal tract was measured using an established method [6, 21, 22]. Briefly, whole tracts were removed, placed on a petri dish and washed with 1 ml of saline. Then, 0.5-ml samples were mixed with 0.5 ml 5% trichloroacetic acid (Sigma-Aldrich) and centrifuged at 10,000 rpm for 5 min in a centrifuge (Sakuma M-160-IV, Tokyo, Japan). The supernatant was mixed with 0.5 ml of 1 M NaOH and absorbance was determined at 550 nm wavelength using a spectrophotometer (DU640, Beckman Coulter, CA, USA).

### Testing for rolling behaviour and fin movements in aquaria

Each intact fish was transferred from a stock tank (10 ppt seawater) to a tank in which a hygroscopic cloth (Willson, Japan) containing 10 ppt seawater was placed to allow each mudskipper to moisten the skin. Mudskippers was also allowed to hold buccal water during the experiment. The humidity in the room was controlled at >80% (high) or <40% (low) by a humidifier (EE-RB, Zojirushi, Japan) and a dehumidifier (CV-P120, Sharp, Japan). The humidity was constantly measured by a hygrometer (W-1, Shinwa measuring tools, Japan). Behaviours were recorded for 30 min. To investigate effects of environmental salinities, mudskippers were transferred to 0 (freshwater), 10, 20 or 30 ppt seawater in the tank. After 24 h, each fish was transferred to an experimental tank with wet cloth containing the corresponding salinity water and behaviours were recorded in <40% humidity.

### Statistics

All summary data are expressed as mean ± standard error of the mean (SEM), with individual data plotted. Statistical significance was determined by paired/unpaired t-test or Wilcoxon signed-rank test; Kruskal-Wallis test followed by a Steel-Dwass post-hoc test; two-way repeated measures ANOVA or a repeated Freidman test followed by a Steel post hoc test as appropriate. All data were checked for normal distributions and equal variances. Kyplot 5.0 (KyensLab, Tokyo, Japan) was used for statistical analysis.

## Results

### Mudskippers exhibited postprandial drinking behaviour on tidal flats

**Fig 1:**
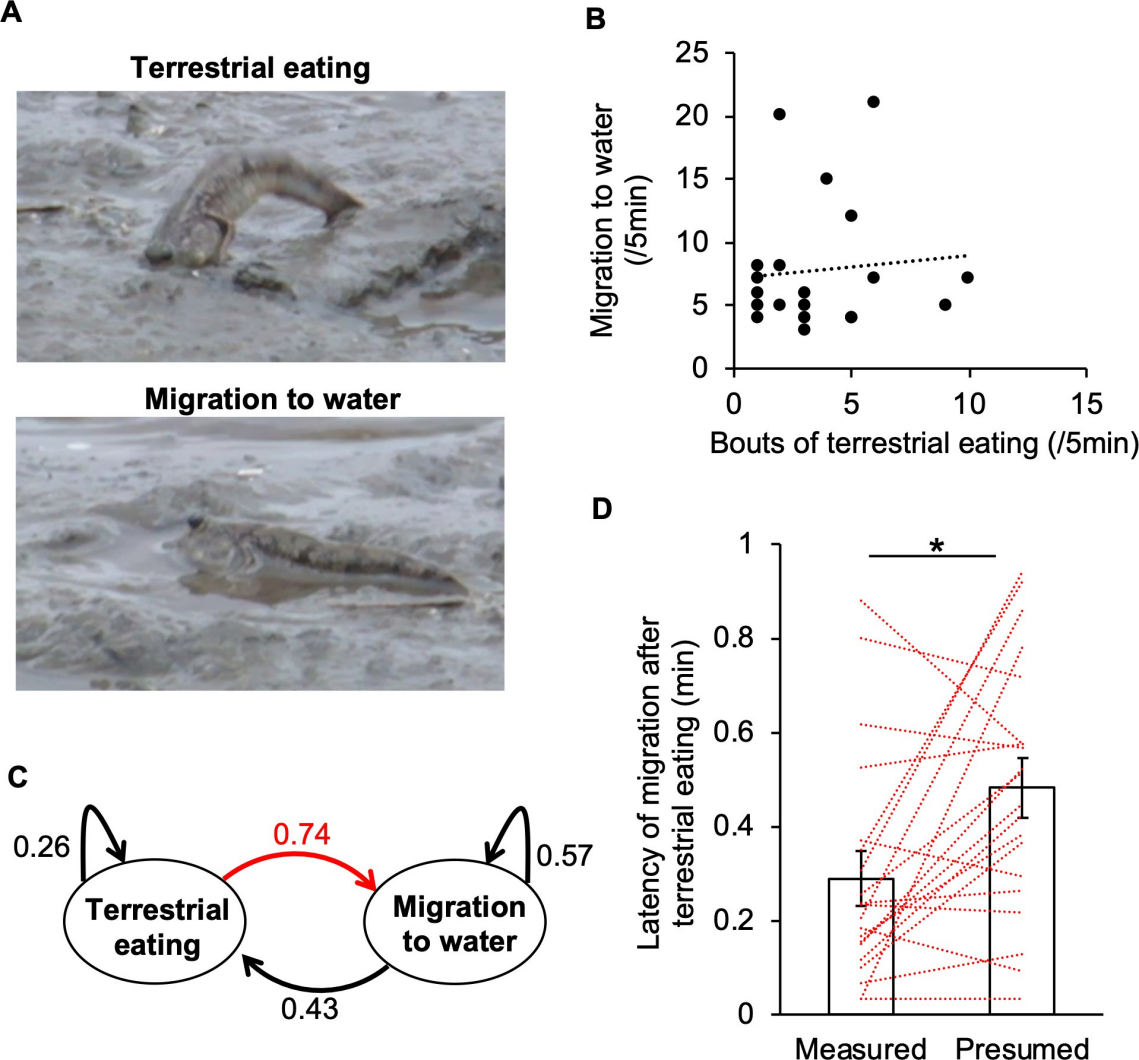
Field observation of postprandial drinking behaviour of mudskippers. (**A**) Example photographs showing terrestrial eating (upper) and migration to water (lower). Terrestrial eating of mudskippers is clearly observed because the fish put its mouth on the mudflat following bending of the body axis. Migration to water areas where mudskippers fully bathed their mouths was counted as an index of a desire to drink. (**B**) Correlation between frequencies of eating bouts and those of migration into water (*n* = 20) in a 5-min observation. (**C**) The behavioural transition between two modes: terrestrial eating and migration into water. Numbers represent transition probabilities calculated from bouts of terrestrial eating (*n* = 46) and migration into water (*n* = 106) from 20 fish. The red arrow shows postprandial drinking. (**D**) Measured and ‘presumed’ latency of migration into a water area after terrestrial eating. The calculation method is illustrated in S1 Fig. *P < 0.01 by paired *t* test. Data are shown as means ± standard error of the mean (SEM) and presented in S1 Table. Paired data from individual fish are shown by dotted lines.

In field observation, each fish on a tidal flat was tracked to evaluate postprandial drinking features (Fig 1A). Correlation between frequencies of terrestrial eating bouts and migration into water was rarely shown (Fig 1B, R^2^ = 0.0083), reflecting many behavioural/physiological factors that induce their migration [5]. To understand the behavioural transition between terrestrial eating and migration into water, the transition probabilities were calculated from ethograms of all fish (Figs 1C and S1). The transition from terrestrial eating to migration was most likely (74%), while the probability of consecutive eating bouts without migration was lower (26%). To confirm a cause-and-effect relationship between terrestrial eating and migration (an index of desire for water), the measured latency of subsequent migration after terrestrial eating was compared with the ‘presumed’ latency on the assumption that migration bouts occur independently of terrestrial eating (S1 Fig). The measured latency was shorter than the ‘presumed’ latency (Fig 1D). Thus, terrestrial eating appeared to lead to migration.

### Postprandial drinking of mudskippers was evoked without systemic dehydration

**Fig 2:**
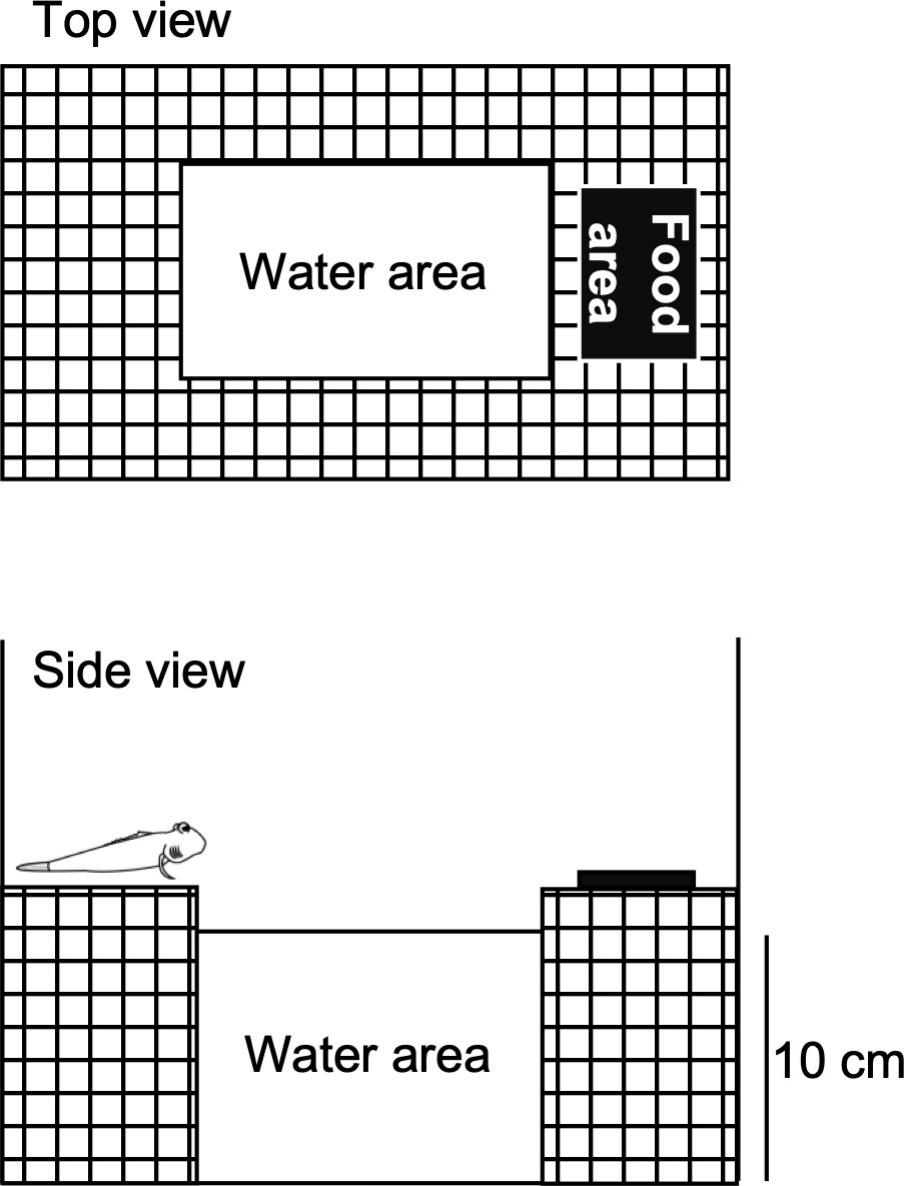
Schematic diagram of the experimental tank used to observe postprandial drinking of mudskippers. 10 ppt seawater is close to the natural environmental salinity and almost identical to the osmolality of body fluids, and thus was chosen for subsequent experiments. Mudskippers were transferred to the tank without food. After 60 min, food was placed on a land area.

**Fig 3:**
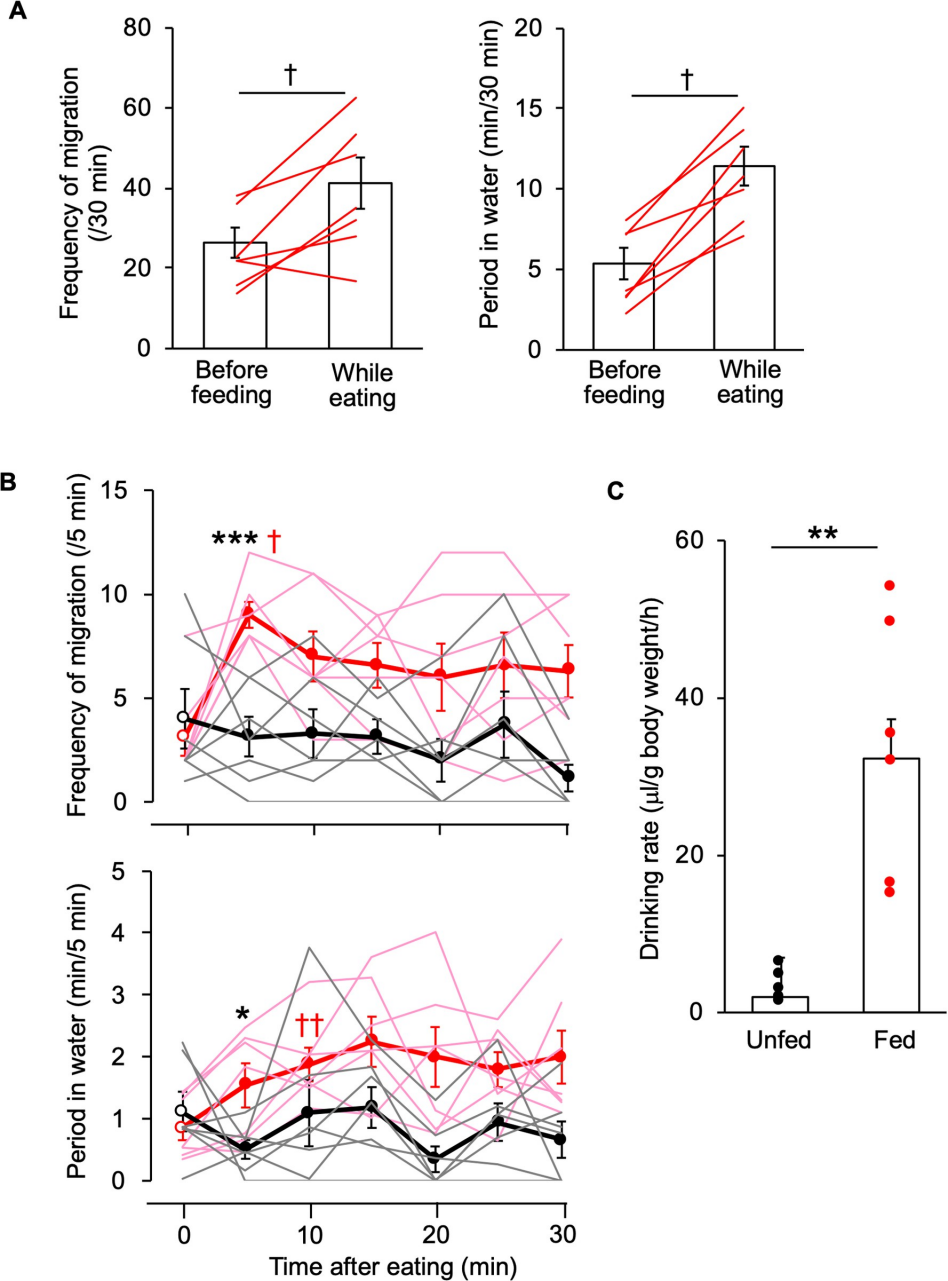
Postprandial drinking behaviour in aquaria. (**A**) Amphibious behaviour before feeding and while eating. The frequency of migration and period of time in water were measured for 30 min. Paired data from individual fish are shown (red lines). A paired Wilcoxon signed-rank test was used for statistical analysis. † *P* < 0.05 vs. controls before feeding. Raw data are presented in S2 Table. (**B**) Effects of eating on time-course changes in amphibious behaviour. Data from fed fish (red lines) and unfed fish (black lines) are expressed as mean ± SEM with individual data. The parameters were measured at 5 (0–5), 10 (5–10), 15 (10–15), 20 (15–20), 25 (20–25), and 30 (25–30) min after onset of eating, as well at 0 (0–5) min (before feeding). Two-way repeated measures ANOVA and a Steel post-hoc test were used for statistical analysis. *P < 0.05, *** *P* < 0.001 vs. unfed controls. †*P* < 0.05, ††*P* < 0.01 vs. controls before feeding. (**C**) Effect of eating on drinking rate. An unpaired Wilcoxon signed-rank test was used for statistical analysis. **P < 0.005 vs. unfed controls. Individual data are plotted with the mean ± SEM and presented also in S3 Table.

Our observation of amphibious behaviour in a tank with dry food on a land area (Fig 2) showed that the period of time in the water area and the frequency of migration into water were increased relative to those before feeding (Fig 3A). We further analysed time-course changes of the amphibious behaviour in fed/unfed fish (Fig 3B). The frequency of migration and period in the water area were increased by 5–10 min after onset of eating, without changes in plasma sodium levels [fed, 167.0 ± 1.9 mM (*n* = 5) vs. unfed control, 167.0 ± 2.0 mM (*n* = 5), *p* = 0.26 with unpaired *t*-test], which are correlated with osmolality in teleosts [23–25]. Correspondingly, the amount of ingested water was increased in the fed fish (Fig 3C).

### Rolling and related behaviour were facilitated in low humidity

**Fig 4:**
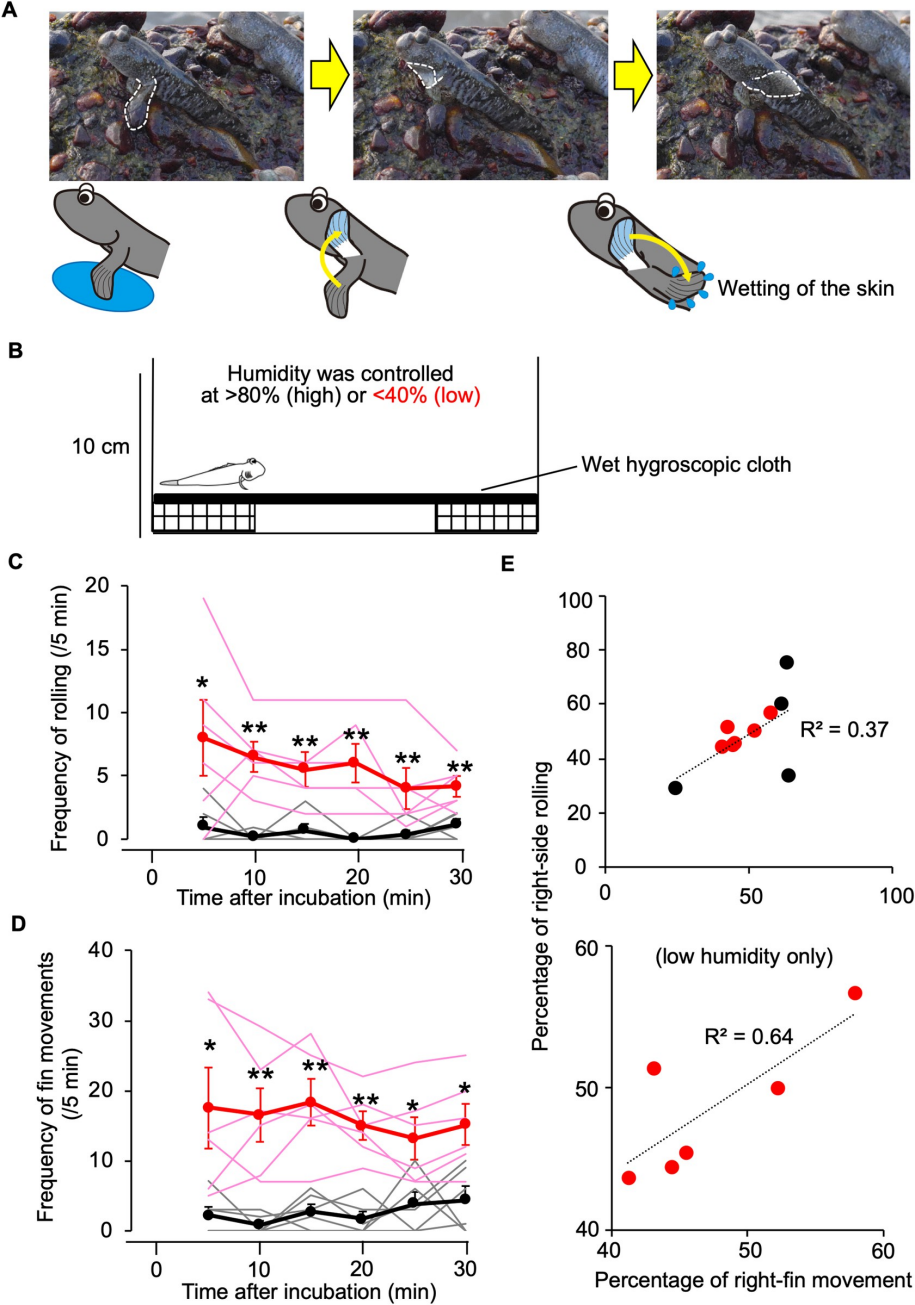
Rolling behaviour and pectoral-fin movements in dry land. (**A**) Photographs showing movements of a pectoral fin (broken lines) with schematic drawings. The pectoral fins touch wet ground (left). The fin sequentially moves forward (middle) and then backward (right). A movie showing movements on dry land is provided in S1 Movie. (**B**) Schematic diagram of the experimental tank used to observe rolling behaviour and pectoral-fin movements in controlled humidity. A wet hygroscopic cloth was placed to allow each mudskipper to moisten the skin. (**C**, **D**) Effects of drying on frequency of rolling behaviour (**C**) and pectoral-fin movements (**D**) after 5 (0–5), 10 (5–10), 15 (10–15), 20 (15–20), 25 (20–25), and 30 (25–30) min. Data from fish in <40% (low) humidity (red lines) and fish in >80% (high) humidity (black lines) are expressed as mean ± SEM with individual data. *P < 0.05, **P < 0.01 by unpaired Wilcoxson-rank test, following a Friedman test. (**E**) Laterality in frequencies of rolling behaviour and pectoral-fin movements. Correlations of percentages of right-side rolling and right fin movements are shown. Black and red circles indicate data for fish in high and low humidity, respectively. R^2^ values were 0.37 using all data (upper panel, *n* = 10) and 0.64 using data from fish in low humidity (lower panel, *n* = 6).

When rolling behaviour was observed in the field, accompanying pectoral-fin movements were often found (Fig 4A). Normally mudskippers put their pectoral fins on the mudflat for ‘walking’ with the fins flexed. During the movement, the flexed fin sequentially moved forward and then extended backward. We hypothesized that both behaviours arise from cutaneous drying. To test this hypothesis, mudskippers were transferred to aquaria with minimum water to allow each mudskipper to moisten the skin (Fig 4B). Both behaviours were more frequent in <40% humidity than in controls in >80% humidity after 5–30 min (Fig 4C and 4D). Since there was no significant difference in weight reduction of mudskippers exposed to 30-min dehydration [<40% humidity, 34.2 ± 5.9 mg (*n* = 6) vs. >80% humidity control, 41.3 ± 11.6 mg (*n* = 6), *p* = 0.93 with Wilcoxson-rank test (S2 Table)], these behaviours appear to be responses to cutaneous drying, rather than to systemic parameters. Simultaneous movements of the left and right fins were not observed. The ratio of left/right rolling was compared with that of ipsilateral fin movements (Fig 4E). A high positive coefficient of laterality was found, particularly in the low humidity group.

**Fig 5:**
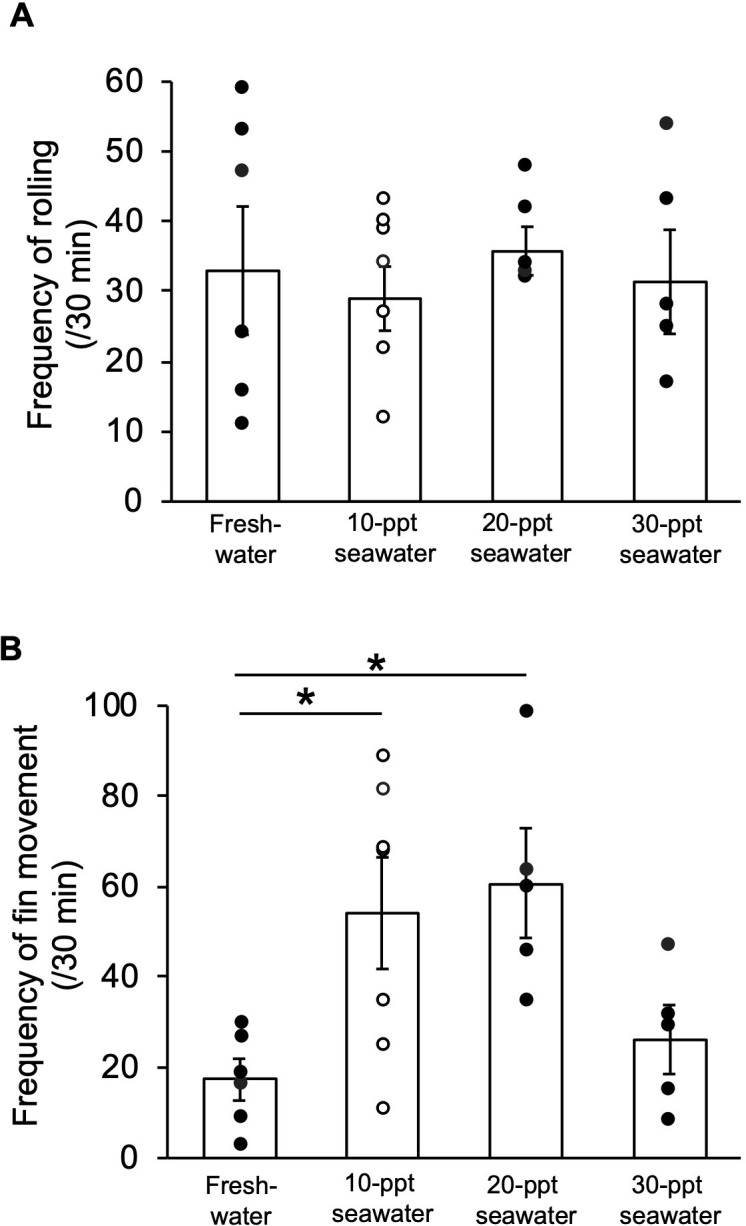
Effects of environmental salinities on rolling and fin movements. (**A**) Rolling behaviour of mudskippers after acclimation to freshwater and 10, 20, and 30 ppt seawater. There were no significant differences by Kruskal-Wallis test (*n* = 5–7). (**B**) Pectoral-fin movements of mudskippers after acclimation to freshwater and 10, 20, and 30 ppt seawater. *P < 0.05 with Kruskal-Wallis followed by Steel-Dwass post-hoc test. Individual data are plotted with the mean ± SEM.

The mudskipper skin, especially the pectoral skin, plays a critical role in transport of ions [26, 27]. Thus, we examined the influence of environmental salinity on rolling and fin movements at low humidity. Rolling behaviour was not significantly affected by environmental salinity (Fig 5A), but fin movements of fish in freshwater were less frequent than those for fish in 10 ppt and 20 ppt seawater (Fig 5B).

## Discussion

**Fig 6:**
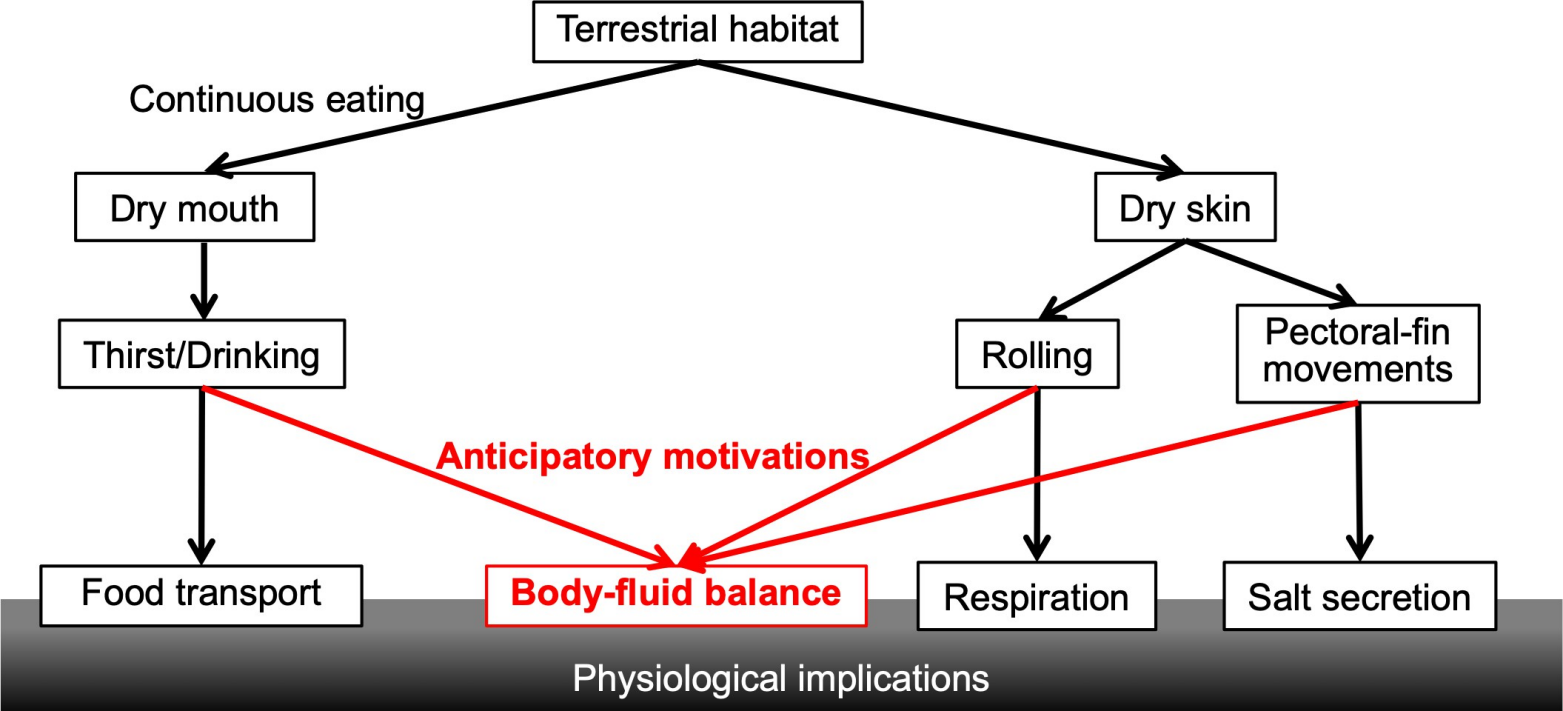
A conceptual diagram of implications of osmoregulatory behaviours in mudskipper fish. In semi-terrestrial mudskippers, peripheral sensations such as a dry mouth following eating and cutaneous drying induces postprandial drinking and rolling behaviour with pectoral-fin movements, respectively. To prevent systemic dehydration, it is likely that behaviours are triggered by anticipatory motivations. In addition, these behaviours may play important roles in transport of terrestrial prey in the digestive tract, cutaneous respiration, and salt secretion. Such multifunctional behaviours may allow mudskippers to survive in terrestrial habitats.

This study shows that peripheral sensation of food and/or prandial drying in the buccal cavity evokes drinking behaviours in semi-terrestrial fish. Since postprandial drinking was also found in mammals and birds [8–10, 28], it might be conserved among (semi-)terrestrial vertebrates although other terrestrial fishes have not been investigated. This postprandial drinking should help animals to prevent future dehydration by food absorption. Furthermore, we suggest that rolling and fin movements of mudskippers occur to maintain skin moisture through peripheral sensation before significant systemic dehydration. Postprandial drinking, rolling, and fin movements also have roles in other physiological processes, including in transport of terrestrial food in the digestive tract [12], cutaneous respiration [4], salt secretion through the skin [20]. These multifunctional behaviours may also allow mudskippers to survive in terrestrial environments (Fig 6).

### Prandial drinking allows mudskippers to extend an overland excursion for terrestrial eating

The postprandial drinking behaviour of mudskippers, which occurred immediately without changes in plasma sodium/osmolality, may stem from a buccal sensation. In mammals, the peripheral sensation of food activates thirst neurons to induce drinking behaviour before any changes in blood parameters [9]. Postprandial drinking in mudskippers also appears to prevent future dehydration induced by ingestion of salty or dry foods [11, 29]. In aquatic fish (rainbow trout), ingested dry diets induced copious drinking, suggesting ‘gastric drying’ [30]. Thus, similar mechanisms of peripheral dry sensing and subsequent drinking regulation may be conserved among vertebrates. In addition to this osmoregulatory role, ingestion of water is required for suction feeding (food transport from the mouth to esophagus) [30, 31]. The mudskipper can swallow terrestrial food without migration into water as long as buccal water is held, whereas some fish species that capture terrestrial food do not hold water in the buccal cavity and must return to water immediately for suction feeding [31, 32]. When mudskippers use up buccal water for eating, they appear to sense buccal drying, which has been defined as thirst in mudskippers [6]. This behavioural feature allows mudskippers to extend an overland excursion for terrestrial eating.

### Unique behaviours of mudskippers may prevent systemic dehydration during land invasion

Rolling and pectoral-fin movements may also arise from peripheral sensation, rather than systemic dehydration. These behaviours are frequently observed in low humidity, which suggests that cutaneous drying leads to behaviours for moistening the skin. Indeed, rolling behaviour in another mudskipper species, *B*. *boddaerti*, has been shown to be induced by a water level decrease [15], and neither rolling nor fin movements were seen in water in our experiments. We preliminary found that the rolling was also induced by injection of an irritant to the dorsal surface, supporting the causal relationship between their cutaneous sensation and rolling behaviour. Coefficient laterality between rolling and fin movements suggests that mudskippers recognize right/left drying sensation in the dorsal surface. Just before the fin movements, the pectoral fins usually touched the wet ground. In our field observation, buccal water leaked from the gill slit during fin movements (see a movie in S1 Movie). Mudskippers on dry ground seem to dip their pectoral fin to buccal water to moisten the dorsal skin. These behaviours may prevent probable systemic dehydration following water loss through the surface [33]. Unlike the rolling, the pectoral-fin movements seem to moisten pits of pectoral fins specifically, in which mudskipper ionocytes (the site of salt secretion) are primarily found [26, 34, 35]. Mudskippers after acclimation to freshwater have significantly decreased pectoral-fin movement frequency. Because ion transport requires water media, the lower frequency of the movements after freshwater adaptation may reflect inhibition of salt secretion thorough ionocytes on the pectoral skin [26, 36], which is triggered by environmental-water ions, rather than plasma parameters [37]. These data suggest the importance of peripheral hydromineral sensing of mudskippers that live in fluctuating environments. By contrast, rolling behaviour is independent of environmental salinities, suggesting its primary role in cutaneous respiration, rather than in ion transport. The semi-terrestrial mudskipper breathes mostly in air [38] and the water layer on the skin surface is essential for oxygen/CO_2_ transport [39]. High degrees of vascularization and permeability of the dorsal surface in mudskippers [40, 41] also support a role for the wet dorsal skin in air breathing.

## Conclusion

We have found repeated evolution of ‘thirst induced by dry mouth’ in the mudskipper taxa. Similarly, rolling might be mediated by an ‘itch sensation induced by dry skin’ in mudskippers as a counterpart of scratching behaviour in mammals [42, 43]. Further analyses may reveal the relationships between habitats and dry skin itch, as well as the unknown evolution of hydromineral sensing [44, 45] during vertebrate terrestrialisation.

## Supporting information

S1 Fig Schematic diagram showing the measured and presumed latency of migration to water following terrestrial eating. Upper panel: measured latency of migration into water. Red circles and blue triangles indicate bouts of terrestrial eating and migration, respectively. Lower panel: calculation of ‘presumed’ latency. The bouts of migration are redistributed on the assumption that migration bouts occur at equal intervals independently of terrestrial eating.
(TIF)

S1 Table Five minutes observation of postprandial drinking behaviour on mudflat. (DOCX)

S2 Table Amphibious behaviour of captive mudskippers before feeding, after food placement and while eating (i.e. after eating onset). (DOCX)

S3 Table Drinking rate after terrestrial eating. Duplicate samples were measured to calculate the average.
(DOCX)

S4 Table Body-weight reduction of mudskippers exposed to 30-min dehydration. (DOCX)

S1 Movie A movie showing pectoral fin movements by the mudskipper on dry land. Buccal refilling and subsequent fin movements were observed on dry land. When the fin moves forward, buccal water leaked from the gill slit. Mudskippers seem to dip their pectoral fin to buccal water to moisten the dorsal skin.
(MP4)
