# Supplementary figures and images for: Behavioural osmoregulation during land invasion in fish: Prandial drinking and wetting of the dry skin
Source: PLoS One. 2022 Dec 7;17(12):e0277968. doi: 10.1371/journal.pone.0277968 (PMC9728915; doi:10.1371/journal.pone.0277968)

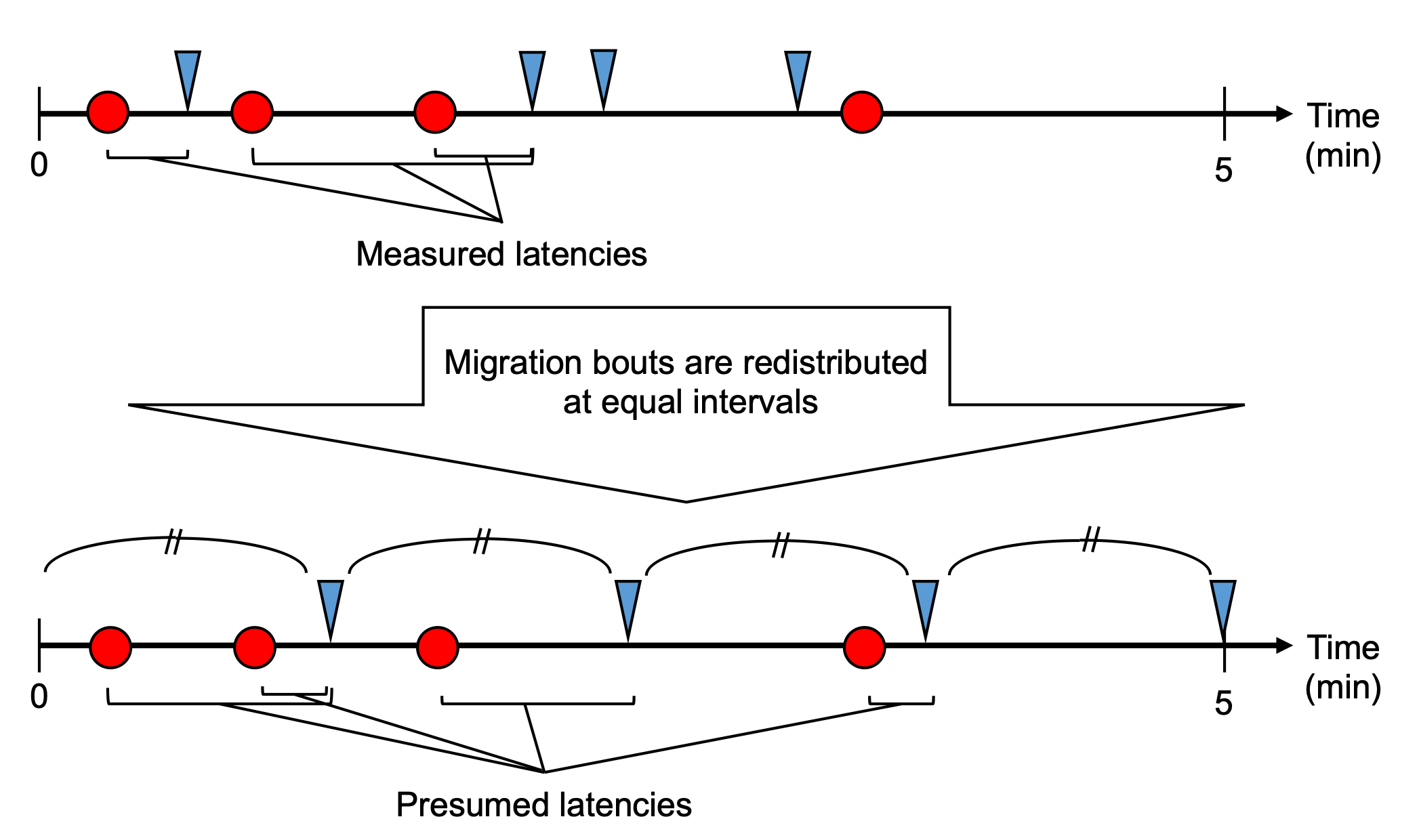

Supplement: S1 Fig — Upper panel: measured latency of migration into water. Red circles and blue triangles indicate bouts of terrestrial eating and migration, respectively. Lower panel: calculation of ‘presumed’ latency. The bouts of migration are redistributed on the assumption that migration bouts occur at equal intervals independently of terrestrial eating. (TIF) [file pone.0277968.s001.tif]
