# Supplementary material for: Behavioural osmoregulation during land invasion in fish: Prandial drinking and wetting of the dry skin
Source: PLoS One. 2022 Dec 7;17(12):e0277968. doi: 10.1371/journal.pone.0277968 (PMC9728915; doi:10.1371/journal.pone.0277968)
Supplement: S1 Table — (DOCX) [file pone.0277968.s002.docx]

| Animal ID | Frequency  of eating | Frequency  of migration | Presumed interval of migration (s) | Measured latency  (s) | Presumed latency  (s) |
| --- | --- | --- | --- | --- | --- |
| Fish 1 | 3 | 3 | 100.0 | 21.0 | 55.3 |
| Fish 2 | 5 | 4 | 75.0 | 22.2 | 17.6 |
| Fish 3 | 3 | 4 | 75.0 | 12.3 | 51.7 |
| Fish 4 | 5 | 4 | 75.0 | 18.4 | 56.6 |
| Fish 5 | 1 | 5 | 60.0 | 10.0 | 23.0 |
| Fish 6 | 2 | 20 | 15.0 | 11.0 | 5.5 |
| Fish 7 | 6 | 21 | 14.3 | 4.0 | 7.7 |
| Fish 8 | 2 | 5 | 60.0 | 7.0 | 25.5 |
| Fish 9 | 1 | 7 | 42.9 | 9.0 | 31.4 |
| Fish 10 | 1 | 4 | 75.0 | 2.0 | 47.0 |
| Fish 11 | 1 | 8 | 37.5 | 48.0 | 43.0 |
| Fish 12 | 1 | 6 | 50.0 | 2.0 | 2.0 |
| Fish 13 | 2 | 8 | 37.5 | 31.5 | 34.5 |
| Fish 14 | 3 | 6 | 50.0 | 6.0 | 22.0 |
| Fish 15 | 6 | 7 | 42.9 | 15.3 | 30.9 |
| Fish 16 | 3 | 5 | 60.0 | 37.0 | 34.0 |
| Fish 17 | 10 | 7 | 42.9 | 9.3 | 26.9 |
| Fish 18 | 9 | 5 | 60.0 | 52.8 | 34.4 |
| Fish 19 | 5 | 12 | 25.0 | 14.2 | 15.8 |
| Fish 20 | 4 | 15 | 20.0 | 14.0 | 13.0 |
