# Supplementary material for: Behavioural osmoregulation during land invasion in fish: Prandial drinking and wetting of the dry skin
Source: PLoS One. 2022 Dec 7;17(12):e0277968. doi: 10.1371/journal.pone.0277968 (PMC9728915; doi:10.1371/journal.pone.0277968)
Supplement: S2 Table — (DOCX) [file pone.0277968.s003.docx]

|  | Before feeding (/30 min) | | After food placement (/30 min) | | While eating (/30 min) | |
| --- | --- | --- | --- | --- | --- | --- |
| Animal ID | Period in water  (min) | Frequency  of migration | Period in water  (min) | Frequency  of migration | Period in water  (min) | Frequency  of migration |
| Fish 21 | 7.6 | 40 | 10.8 | 51 | 10.4 | 50 |
| Fish 22 | 3.6 | 24 | 15.6 | 29 | 13.0 | 19 |
| Fish 23 | 7.6 | 24 | 15.5 | 30 | 15.5 | 30 |
| Fish 24 | 3.7 | 18 | 11.8 | 31 | 11.2 | 34 |
| Fish 25 | 4.0 | 38 | 6.6 | 62 | 7.5 | 64 |
| Fish 26 | 2.6 | 25 | 10.2 | 53 | 8.4 | 55 |
| Fish 27 | 8.5 | 16 | 12.7 | 37 | 14.1 | 37 |
