# Supplementary material for: Behavioural osmoregulation during land invasion in fish: Prandial drinking and wetting of the dry skin
Source: PLoS One. 2022 Dec 7;17(12):e0277968. doi: 10.1371/journal.pone.0277968 (PMC9728915; doi:10.1371/journal.pone.0277968)
Supplement: S3 Table — Duplicate samples were measured to calculate the average. (DOCX) [file pone.0277968.s004.docx]

| Animal ID | Treatment | Drinking rate  (μl/fish/h) | Body Weight (g) | Drinking rate (μl/g BW/h) |
| --- | --- | --- | --- | --- |
| Fish 28 | Non feeding | 16.9 | 4.80 | 3.5 |
| Fish 29 | Non feeding | 0.4 | 4.26 | 0.1 |
| Fish 30 | Non feeding | 12.0 | 7.49 | 1.6 |
| Fish 31 | Non feeding | 1.6 | 2.85 | 0.6 |
| Fish 32 | Non feeding | 2.6 | 2.54 | 1.0 |
| Fish 33 | Non feeding | 13.5 | 2.70 | 5.0 |
| Fish 34 | Feeding | 64.0 | 4.23 | 15.1 |
| Fish 35 | Feeding | 52.3 | 3.78 | 13.8 |
| Fish 36 | Feeding | 129.3 | 2.70 | 47.9 |
| Fish 37 | Feeding | 160.3 | 3.05 | 52.5 |
| Fish 38 | Feeding | 98.9 | 3.26 | 30.3 |
| Fish 39 | Feeding | 61.1 | 1.80 | 33.9 |
