# Supplementary material for: Behavioural osmoregulation during land invasion in fish: Prandial drinking and wetting of the dry skin
Source: PLoS One. 2022 Dec 7;17(12):e0277968. doi: 10.1371/journal.pone.0277968 (PMC9728915; doi:10.1371/journal.pone.0277968)
Supplement: S4 Table — (DOCX) [file pone.0277968.s005.docx]

| Animal ID | Treatment | Decrease in BW (g) |
| --- | --- | --- |
| Fish 40 | High humidity | 0.023 |
| Fish 41 | High humidity | 0.040 |
| Fish 42 | High humidity | 0.036 |
| Fish 43 | High humidity | 0.040 |
| Fish 44 | High humidity | 0.019 |
| Fish 45 | High humidity | 0.091 |
| Fish 46 | Low humidity | 0.031 |
| Fish 47 | Low humidity | 0.040 |
| Fish 48 | Low humidity | 0.020 |
| Fish 49 | Low humidity | 0.040 |
| Fish 50 | Low humidity | 0.020 |
| Fish 51 | Low humidity | 0.054 |
